# Double defects-induced elastic wave coupling and energy localization in a phononic crystal

**DOI:** 10.1186/s40580-021-00277-4

**Published:** 2021-09-16

**Authors:** Soo-Ho Jo, Yong Chang Shin, Wonjae Choi, Heonjun Yoon, Byeng D. Youn, Miso Kim

**Affiliations:** 1 Department of Mechanical Engineering, Seoul National University, 08826 Seoul, Republic of Korea; 2 Institute of Advanced Machines and Design, Seoul National University, 08826 Seoul, Republic of Korea; 3 AI Metamaterial Research Team, Korea Research Institute of Standards and Science, 34113 Daejeon, Republic of Korea; 4 School of Mechanical Engineering, Soongsil University, 06978 Seoul, Republic of Korea; 5 OnePredict Inc, 06160 Seoul, Republic of Korea; 6 School of Advanced Materials Science & Engineering, Sungkyunkwan University, 16419 Suwon, Republic of Korea

**Keywords:** Phononic crystal, Energy localization, Defect band splitting, Double defect modes

## Abstract

This study aims to investigate elastic wave localization that leverages defect band splitting in a phononic crystal with double defects through in-depth analysis of comparison of numerical and experimental results. When more than one defect is created inside a phononic crystal, these defects can interact with each other, resulting in a distinctive physical phenomenon from a single defect case: defect band splitting. For a phononic crystal consisting of circular-hole type unit cells in a thin aluminum plate, under A_0_ (the lowest antisymmetric) Lamb waves, both numerical simulations and experiments successfully confirm the defect band splitting phenomenon via frequency response functions for the out-of-plane displacement calculated/measured at the double defects within a finite distance. Furthermore, experimental visualization of in-phase and out-of-phase defect mode shapes at each frequency of the split defect bands is achieved and found to be in excellent agreement with the simulated results. Different inter-distance combinations of the double defects reveal that the degree of the defect band splitting decreases with  the increasing distance due to weaker coupling between the defects. This work may shed light on engineering applications of a multiple-defect-introduced phononic crystal, including broadband energy harvesting, frequency detectors, and elastic wireless power transfer.

**Supplementary Information:**

The online version contains supplementary material available at 10.1186/s40580-021-00277-4.

## Introduction

Phononic crystals are artificial periodic structures that can efficiently tailor propagation of acoustic or elastic waves in solids [1, 2]. The first phononic crystal concept dates back to 1993 when Kyshwaha et al. [3] presented the first acoustic band structure of periodic elastic composites, drawing the analogy to electromagnetic waves of the photonic crystal concept for light. Phononic crystals have drawn a great deal of attention in the research community because of both their unusual and extraordinary physical properties that cannot be exhibited by natural materials [4, 5], and their potential applications.

A phononic band gap, which refers to a certain frequency range within which no acoustic or elastic waves can propagate through the  phononic crystal [6], is one of the important and unique wave controlling features of phononic crystals along with negative refraction [7, 8], wave self-collimation [9, 10], wave focusing [11, 12], and cloaking [13, 14]. The most common physical mechanism for phononic band gap formation is Bragg scattering [15, 16], whereas the existence of local resonance [17, 18] contributes to the band gap of acoustic or elastic metamaterials. The Bragg scattering-type band gap is a result of destructive interferences induced by the periodicity of unit cells when wavelengths are on the scale of the lattice constant of a unit cell. For the past years, development of numerical methods for designing phononic band gaps having a desired frequency range has thus been the major research focus in the phononic crystal realm by modulating properties of unit cells. In this regard, the effects of unit cells' properties such as shapes [19, 20], geometric dimensions [21, 22], and material properties [23, 24] on the band gap performance has been intensively investigated in a number of studies. Phononic band gaps can provide quite a versatile platform for designing wave functionalities such as mirrors [25, 26], cavities [27, 28], and waveguides [29, 30], opening up new avenues in a variety of engineering applications, including noise control [31, 32], vibration reduction [33, 34], and waveguiding [35, 36].

Indeed, creating a defect in a perfect phononic crystal is a powerful way to realize wave energy localization in acoustic or elastic domains. Wave energy localization that leverages a defect mode of a phononic crystal has gained substantial attention due to its capabilities of confining and amplifying wave energy at the desired point. When a single defect is created in a phononic crystal by locally breaking the periodicity of unit cells, such as by replacing a unit cell with another structure having different material or geometry, flat passbands (*i.e.,* defect bands) are generated within a phononic band gap [37, 38]. When acoustic or elastic waves are generated in a phononic crystal system near a defect band frequency, they can become localized inside the defect while representing a certain defect mode shape [39, 40]. Defect-mode-enabled energy localization has proven highly desirable for several potential applications, such as energy harvesting [20, 41], wave filtering [42, 43], and sensors [44, 45].

Recently, several research groups have also turned their attention to the case where more than one defect is present inside a phononic crystal. A few studies on coupling of defect modes in a phononic crystal with double or multiple defects have been made for the past decade in both acoustic and elastic regimes. In acoustic domains, several works in Ref. [46–49] showed that the evanescent modes localized in the double defects interact with each other and generate their splitting of defect bands when the double defects are created sufficiently close to each other along the incident wave direction inside a phononic crystal. Phononic crystal systems in each prior work differ in their physical nature of the inclusion and the matrix: water cylinders in mercury background [46], aluminum cylinders in air [47], steel cylinders in water [48], and PVC cylinders in air [49]. The latter two cases include both numerical prediction and experimental demonstration while the first two cases covered only numerical analysis. In elastic domains, Korovin et al. [50] numerically observed the defect band splitting phenomenon in a one-dimensional phononic crystal with double defects. Jo et al. [51] numerically explored the possibilities of piezoelectric energy harvesting that leveraged the double defect modes in a two-dimensional phononic crystal with double defects. More recently, Geng et al. [52] investigated the influence on the defect coupling behavior and energy harvesting in a phononic crystal beam with double defects with numerical simulations. Although a few numerical studies have been reported on double defect modes in a phononic crystal, further understanding on double defect modes in elastic domain based on comparison of results determined by numerical simulations and experiments is still required.

Hence, the research herein aims at not only numerically analyzing but also experimentally validating various aspects of wave physics in double-defected phononic crystals, including defect band splitting phenomenon and defect mode shapes. A two-dimensional phononic crystal consisting of circular hole-type unit cells with either a single defect or double defects having different finite distances is used under A_0_ (the lowest antisymmetric) Lamb waves. We further delve into the effects of the distance between the double defects on the degree of the defect band splitting via both numerical simulations and experiments. An experimental setup leads to a successful demonstration of defect band splitting and visualization of various defect mode shapes at each frequency of split defect bands, all of which are found in great agreement with numerical analysis results. Furthermore, comparison of numerical and experimental results is elucidated in terms of boundary conditions and physical uncertainties of material properties and geometry.

## Materials and methods

### Single or double defects-introduced phononic crystal design

**Fig. 1:**
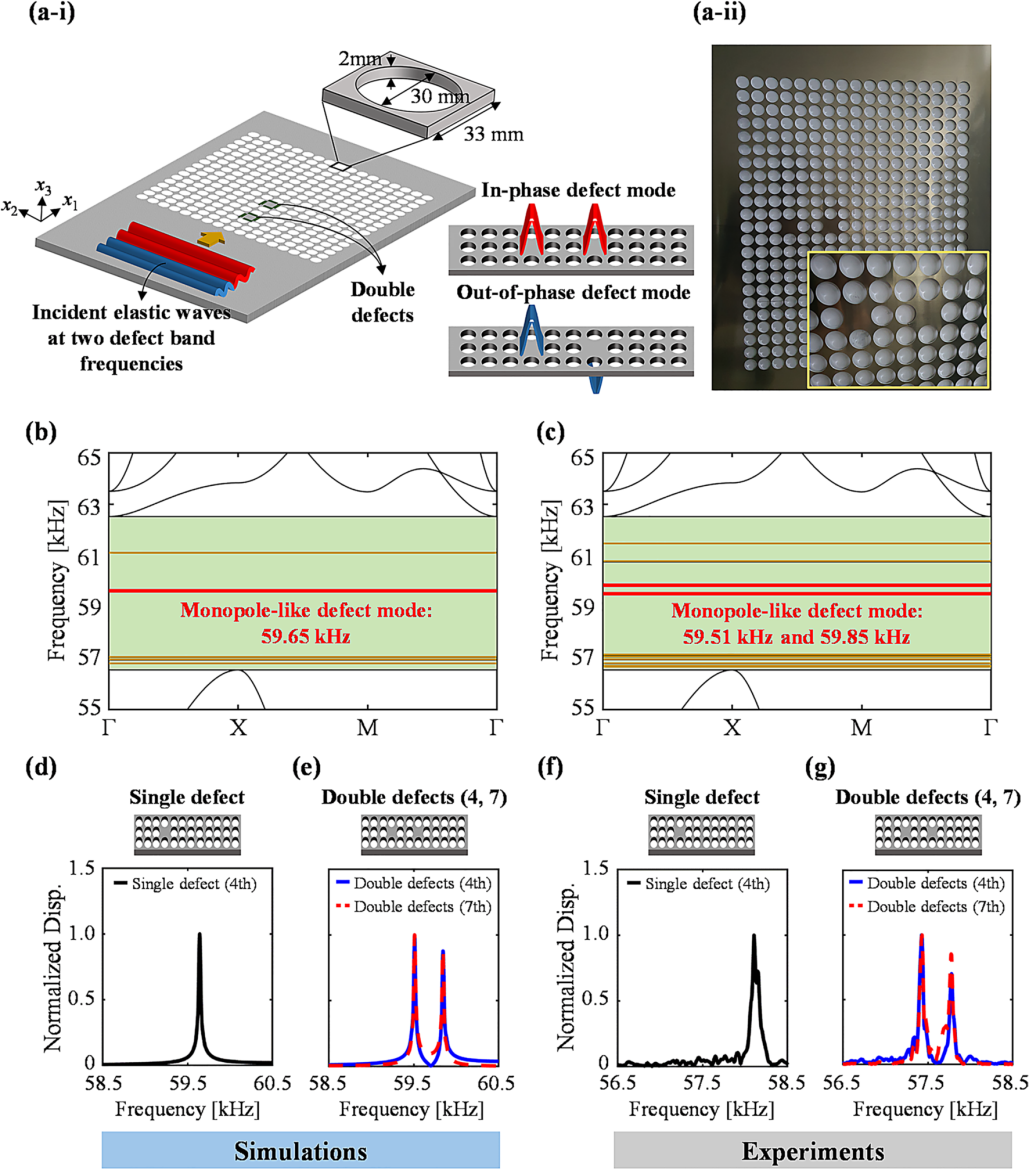
Numerical simulation and experiment results of elastic wave localization that leverages the defect band splitting phenomenon of a phononic crystal with double defects (4, 7). **a** A schematic illustration (left) of in-phase and out-of-phase localized behaviors at each frequency of split defect bands and a snapshot (right) of one fabricated specimen. **b** Formation of five defect bands in the band gap, ranging from 56.55 kHz to 62.51 kHz, and the highlighted target defect band of 59.65 kHz presenting the monopole-like defect mode shape. **c** Splitting of the five defect bands presented in **b** into ten defect bands due to coupling between the double defects (4, 7), and the highlighted target defect bands of 59.51 kHz and 59.85 kHz. (**d**, **e**) Numerically and (**f**, **g**) experimentally demonstrated the defect band splitting phenomenon via comparison of the normalized out-of-plane displacement frequency responses functions in the cases of single and double defects (4, 7), respectively

A schematic illustration of the phononic crystal with double defects in a 2 mm-thick aluminum plate used in this work is depicted in Fig. 1a-i, while Fig. 1a-ii shows the fabricated specimen of the designed phononic crystal. The phononic crystal consists of a 17 × 17 array of unit cells and each unit cell is composed of a square lattice with a circular hole at the center. Here, the supercell size of the 17 × 17 array is selected because a sufficient number of unit cells should be arranged to guarantee high accuracy in calculating defect band frequencies [53]. The lattice constant and the thickness of the unit cell, and the radius of the circular hole are 33, 2, and 15 mm, respectively. The mass density, Young’s modulus, and Poisson’s ratio of the aluminum are assumed to be 2700 kg/m^3^, 70 GPa, and 0.33, respectively, in numerical simulations. The reference for these values is Ref. [54]. Defect bands highly rely on the selected material and geometry of a unit cell. In this work, we prefer a unit cell design, which can 1) exhibit energy-localized behaviors with the target defect mode shapes near 60 kHz and 2) represent defect bands corresponding to other defect mode shapes considerably distant from the target defect bands. With these purposes in mind, the presented unit cell is selected after parametric studies.

Three cases of double defects are considered depending on their relative inter-distances. In Fig. 1a, one defect is introduced by not creating the circular hole in the 4th unit cell along the *x*_1_-direction when the closest unit cell is considered as the 1st unit cell in reference to the incident waves. The other defect is then introduced in the same manner at the 1) 7th, 2) 8th, and 3) 9th unit cells to create three different cases of double defects. We denote these three double defect cases by (4, 7), (4, 8), and (4, 9); Fig. 1a corresponds to (4, 7) case. Note that the same phononic crystal with a single defect at the 4th unit cell location is used for comparison throughout this work. All defects are deployed at the centerline along the *x*_2_-direction. The reason for selecting the 4th unit cell as the fixed defect is that it proved to be the desirable location in terms of the highest energy localization performance for a phononic crystal with a single defect in our previous study [39, 55].

### Methods for numerical simulations

To calculate band structures of phononic crystals with single or double defects, a tensor-form of the governing equations in an elastic medium is employed:

1 $$\rho \frac{{\partial^{2} u_{i} }}{{\partial t^{2} }} = \left( {\lambda + \mu } \right)\frac{{\partial^{2} u_{i} }}{{\partial x_{i} \partial x_{j} }} + \mu \frac{{\partial^{2} u_{i} }}{{\partial x_{j}^{2} }}$$

where *ρ*, *λ*, and *μ* are the mass density, the 1st and the 2nd Lame’s constants of the solid, respectively. *u*_i_ is the *x*_i_-directional component of a displacement vector **u**. It is known that Eq. (1) can be separated into two different equations that represent the *x*_1_*x*_2_-mode (longitudinal and transverse waves) and the *x*_3_-mode (transverse wave) when the periodic unit cells are deployed in both *x*_1_ and *x*_2_-axes. For each mode, the equation can be shown as:

2 $$\rho \left[ {\begin{array}{*{20}c} {\frac{{\partial^{2} u_{1} }}{{\partial t^{2} }}} \\ {\frac{{\partial^{2} u_{2} }}{{\partial t^{2} }}} \\ \end{array} } \right] = \left( {\lambda + 2\mu } \right)\left[ {\begin{array}{*{20}c} {\frac{\partial }{{\partial x_{1} }}\left( {\nabla \cdot {\mathbf{u}}} \right)} \\ {\frac{\partial }{{\partial x_{2} }}\left( {\nabla \cdot {\mathbf{u}}} \right)} \\ \end{array} } \right] - \mu \left[ {\begin{array}{*{20}c} {\frac{\partial }{{\partial x_{2} }}\left( {\frac{{\partial u_{2} }}{{\partial x_{1} }} - \frac{{\partial u_{1} }}{{\partial x_{2} }}} \right) - \frac{\partial }{{\partial x_{3} }}\left( {\frac{{\partial u_{1} }}{{\partial x_{1} }} - \frac{{\partial u_{3} }}{{\partial x_{2} }}} \right)} \\ {\frac{\partial }{{\partial x_{3} }}\left( {\frac{{\partial u_{3} }}{{\partial x_{2} }} - \frac{{\partial u_{2} }}{{\partial x_{3} }}} \right) - \frac{\partial }{{\partial x_{1} }}\left( {\frac{{\partial u_{2} }}{{\partial x_{1} }} - \frac{{\partial u_{1} }}{{\partial x_{2} }}} \right)} \\ \end{array} } \right]$$

3 $$\rho \frac{{\partial^{2} u_{3} }}{{\partial t^{2} }} = \left( {\lambda + 2\mu } \right)\frac{\partial }{{\partial x_{3} }}\left( {\nabla \cdot {\mathbf{u}}} \right) - \mu \left[ {\frac{\partial }{{\partial x_{1} }}\left( {\frac{{\partial u_{1} }}{{\partial x_{3} }} - \frac{{\partial u_{3} }}{{\partial x_{1} }}} \right) - \frac{\partial }{{\partial x_{2} }}\left( {\frac{{\partial u_{3} }}{{\partial x_{2} }} - \frac{{\partial u_{2} }}{{\partial x_{3} }}} \right)} \right]$$

Following Floquet-Bloch theorem [56], the periodic boundary condition is applied to the outside surfaces of the phononic crystal as:

4 $${\mathbf{u}}\left( {x_{1} + S_{1} a,\,x_{2} + S_{2} a,\,t} \right) = {\mathbf{u}}\left( {x_{1} ,\,x_{2} ,\,t} \right)\exp \left( {j\left( {k_{1} S_{1} a + k_{2} S_{2} a} \right)} \right)$$

where *S*_i_ refer to the number of unit cells along the *x*_i_-direction. *k*_i_ is *x*_i_-axial component of a Bloch wavevector **k**_Bloch_. From Equations (2)-(﻿4), the assumption of harmonic motions derives an eigenvalue problem as:

5 $$\left( {\left[ {{\mathbf{K}}\left( {{\mathbf{k}}_{{{\text{Bloch}}}} } \right)} \right] - \omega^{2} \left[ {\mathbf{M}} \right]} \right){\mathbf{q}} = {\mathbf{0}}$$

where [**M**] and **q** are the mass matrix and the eigenvector of the phononic crystal, respectively. [**K**(**k**_Bloch_)], a function of a Bloch wavevector **k**_Bloch_, is the stiffness matrix of the phononic crystal. Consequently, the calculated eigenfrequencies *ω* for the Bloch wavevectors **k**_Bloch_ that belong to the 1st irreducible Brillouin zone (Γ → X → M → Γ), as shown in Additional file e 1: Fig. S1, offer dispersion relations. The points Γ, X, and M means (*k*_1_, *k*_2_) = (0, 0), (π/*S*_1_*a*, 0), and (π/*S*_1_*a*, π/*S*_2_a), respectively. From the dispersion curves, phononic band gaps and defect bands can be analyzed. A commercially available software package Comsol Multiphysics 5.5 was employed in this study.

Additiona file 1: Fig. S2 illustrates a schematic view of the aluminum plate where a phononic crystal with double defects (4, 7) is deployed under the infinite domain condition. To investigate energy localization characteristics under unidirectional plane waves, two boundary conditions are applied at boundaries of the plate. One is a perfectly matched layer, highlighted with dark blue, which works as an absorbing boundary condition in the *x*_1_-axis. The other is the periodic boundary condition, highlighted with brown, which ensures the infinite domain in the *x*_2_-axis. The length of the perfectly matched layers is twice wavelength of A_0_ Lamb waves at 60 kHz for the 2 mm-thick aluminum plate; the wavelength is obtained by Rayleigh-Lamb wave equation [57]. Incident A_0_ Lamb waves are induced by transversely loading the cross-section of the plate, expressed as a black line, with a prescribed displacement. The distance between the front of the circular holes in the 1st layer and the excitation area is 100 mm, which is larger than five times the wavelength of the A_0_ Lamb waves at 60 kHz. In this study, a hexahedral element with a quadratic serendipity shape function is used as a discretization scheme; the maximum mesh size is less than one-tenth of the wavelength at 60 kHz. These conditions are consistently applied into all plates. There are two important physical quantities for analyzing defect-mode-enabled energy localization: 1) the frequency response function for the out-of-plane displacement calculated at the center of the defects and 2) the displacement field inside and in the vicinity of the defects at each defect band frequency.

Additional file 1: Fig. S3 presents a top view of the large aluminum plate with the same phononic crystal under the finite domain condition. The length and height of the aluminum plate are 2000 and 1000 mm, respectively. The distance between the left end of the plate and circular holes in the 1st layer is 1000 mm. Traction-free conditions are applied to all surfaces of the plates. When incident elastic waves enter into the 1st layer, they will be reflected at the interfaces of the aluminum plate and a set of holes due to the impedance mismatching. Then, the reflected waves will propagate toward the edges of the plate and be reflected at the edges. After the repetitive reflections at mechanical-impedance-mismatched interfaces within the plate, the out-of-plane displacements fields of the entire structure are provided. Note that the same mesh setting is applied in the finite condition case to guarantee consistency with the infinite condition case.

### Methods for experiments

We fabricate four large aluminum plates (2000 × 1000 ×  2 mm^3^) for 17 × 17 phononic crystal supercell specimens with a single and three double defect combinations. To mimic the infinite domain condition in numerical simulations, the plates are made as large as possible while considering the available volume of the experiment room. Next, the computer-aided design files of the phononic crystals with single or double defects are made by using Solidworks 2019. Following an inserted computer-aided design file, a laser-cutting machine fabricates the phononic crystal on the previously prepared aluminum plate. For all plates, a set of circular holes with the double defects is created to be biased to the center of the plate along the *x*_1_-axis, while deployed in the center along the *x*_2_-axis. Note that no polishing is recommended to guarantee the high-intensity of the optical laser signal when measuring with scanning laser Doppler vibrometer.

Additional file 1: Fig. S5a, b illustrate a schematic and a photo of the experimental setup along with the fabricated aluminum plate, respectively. The experimental setup consists of 1) elastic wave generation system (*i.e.*, disc-type lead zirconate titnanate (PZT) transducer (Cerocomp Co.), function generators (Keysight 33512B), and power amplifier (AE Techron 7224)) and 2) visualization system (*i.e.*, laser Doppler vibrometer, data acquisition (DAQ), and controller (Polytec, PSV-400, OFV-5000)). To observe frequency response functions for out-of-plane displacements measured at the center of defects, frequency-modulated continuous-waves are induced with ramp input signals in broadband frequencies ranging from 55 to 60 kHz through the elastic wave generation system. Moreover, to visualize mechanical behaviors of defects, we scan a square area (30 × 30 mm^2^) within the single or double defects while generating continuous-wave signals at certain peak frequencies in frequency response functions for the displacement. Only one area is scanned at one frequency for the single defect case, while two areas are scanned at two frequencies for the double defect case. Note that it is desirable to transfer constant amount of mechanical energy to the defected phononic crystals, regardless of the operating input frequency, in order to clearly observe and investigate double defects-induced energy localization and amplification at defect band frequencies. Hence, we adopt a resonant-type transducer used for 50 kHz, which is sufficiently away from the frequency domain of interest (*i.e.*, from 55 to 60 kHz). In the preliminary experiment, we confirmed that the amplitude of the generated A_0_ Lamb waves was almost constant in this frequency range.

In the cases of the single defect and double defects (4, 7), Additional file 1: Fig. S6a–c present the results of the out-of-plane displacements measured at the central point of the defects in the time domain, respectively. The sampling frequency is 5.12 MHz and the frequency sweeping time is 204.8 ms. Next, we perform fast Fourier transform into these data. With the given sampling frequency and time range, the frequency resolution is near 10 Hz. The frequency response function for the displacement is normalized with the maximum value in each case. These kinds of data are also measured for the other specimens.

## Results and discussion

Figure 1b, c show the calculated band structures for the 17 × 17 supercell with a single defect and double defects (4, 7), respectively. Regardless of the number of defects, the same phononic band gap emerges from 56.55 to 62.51 kHz, colored in a light-green box in Fig. 1b, c. Within the band gap, five distinctive defect bands appear for a single defect case in Fig. 1b. Defect band frequencies are outlined as 56.83, 56.87, 56.94, 59.65, and 61.00 kHz. In the double defect case, each defect band from the single defect undergoes splitting of defect bands into two, resulting in total number of ten distinctive defect bands within the band gap, as shown in Fig. 1c. Defect band frequencies are {56.80, 57.06 kHz}, {56.71, 57.12 kHz}, {56.86, 57.16 kHz}, {59.51, 59.85 kHz}, {60.75, and 61.45 kHz}. For convenience, two split frequencies derived from the same single frequency are paired in parenthesis. Such splitting of defect bands is the result of the coupling of the evanescent modes localized in the double defects. When elastic waves propagate through the phononic crystal with the single defect at one of the defect band frequencies, the single defect behaves as a single mechanical resonator, thus localizing the elastic waves inside the defect while exhibiting the corresponding characteristic defect mode shape (Additional file 1: Fig. S4). Meanwhile, since the double defects are physically connected via the aluminum plate medium (unit cells) located between them, the double defects can be simply mimicked to two mechanical resonators connected with a spring in-between. Thus, the coupling can be regarded as a spring between two resonators, and its degree depends on the distance between the defects. This interaction creates the splitting of defect bands. It should be noted that the defect band splitting is the phononic crystal system’s characteristics not affected by the incident waves.

To more clearly see the defect band splitting phenomenon, the frequency response functions for out-of-plane displacements are numerically calculated at the center of each defect for single and double defect (4, 7) cases in Fig. 1d, e, respectively. Detailed settings for time-harmonic analysis are described in Sect. 2.2. A single peak at 59.65 kHz shown in a single-defected phononic crystal in Fig. 1d undergoes splitting and yields two distinctive peaks of 59.51 kHz and 59.85 kHz in Fig. 1e, as a result of the interaction between the 4th and 7th defects. Note that among the five defect mode shapes, we focus on the certain defect band frequency of 59.65 kHz because the out-of-plane monopole-like defect mode shape at this frequency, as shown in Additional file 1: Fig. S4, was found to be most advantageous for minimizing voltage cancellation [58, 59] when incorporating defect modes of phononic crystals into energy harvesting applications, as addressed in previous studies [39, 40, 51, 60].

In order to experimentally validate the defect band splitting phenomenon, we fabricated four 17 × 17 phononic crystal supercell specimens each containing a single or three different double defect combinations (4, 7), (4, 8), and (4, 9) in 2 mm-thick aluminum plates via laser cutting. Additional file 1: Fig. S5 demonstrates the illustrative schematic and actual picture of the experimental setups we designed for elastic wave generation and visualization. Further details on experimental characterization are available in Sect. 2.3. The defect band splitting behavior in the double-defected phononic crystal is successfully observed in experimental frequency responses functions for out-of-plane displacements measured at the center of each defect for single and double defect (4, 7) cases in Fig. 1f, g, respectively. Specifically, the single peak at 58.11 kHz in Fig. 1f with a single defect is split into two distinctive peaks at 57.43 and 57.78 kHz in the double defect (4, 7) case in Fig. 1g. Moreover, the quantitative degree of splitting is obtained as approximately 350 Hz in experimental measurement (Fig. 1g), which agrees well with the numerical results in Fig. 1e where the frequency spacing of 340 Hz is found. Considering the fact that the frequency range of interest is around 55–60 kHz, the discrepancy of 10 Hz between simulation and experiment is indeed extremely a small difference. Thus, we can confirm the accuracy of our experimental validation method. It should however be noted that there is a discrepancy of 1.5–2 kHz in the absolute peak frequency values between simulation and experiment (Fig. 1d–g). This comes from the inherent physical uncertainties of material properties and manufacturing tolerance of geometric dimensions [61]. Based on the sensitivity analysis of the defect band frequencies as functions of material and geometric properties, we perform and address in the later part of this article (Fig. 6), the quantitative discrepancy of 1.5–2 kHz has proved to fall within in a reasonable range.

**Fig. 2:**
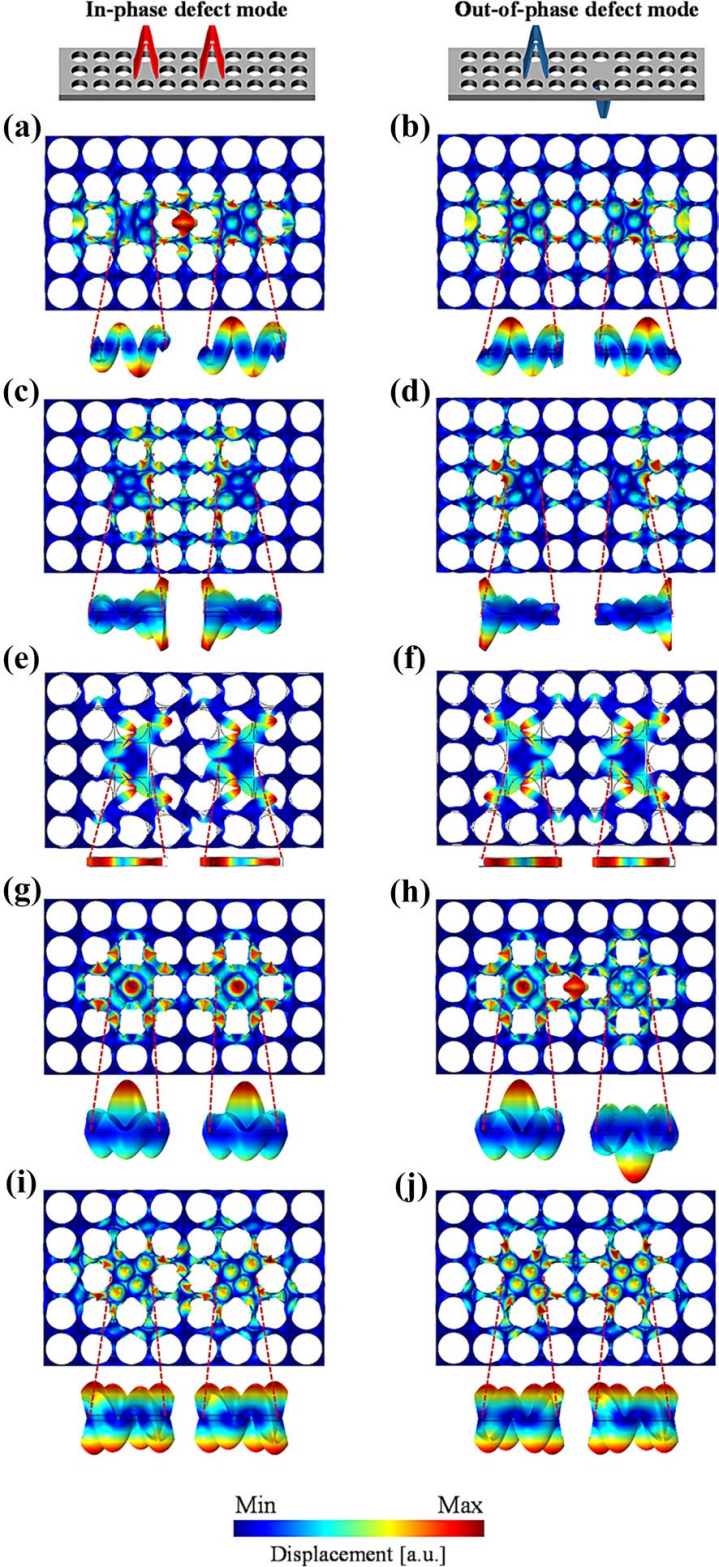
In-phase and out-of-phase defect mode shapes (out-of-plane displacement fields) for the phononic crystal with the double defects (4, 7) at defect band frequencies presented in Fig. 1(c): **a** 56.80 kHz, **b** 57.06 kHz, **c** 56.71 kHz, **d** 57.12 kHz, **e** 56.86 kHz, **f** 57.16 kHz, **g** 59.51 kHz (in-phase monopole-like defect mode shape), **h** 59.85 kHz (out-of-phase monopole-like defect mode shape), **i** 60.75 kHz, and **j** 61.45 kHz

In order to better understand the wave behaviors at each defect band, all the defect mode shapes inside the defects of the (4, 7) double-defected phononic crystal are numerically achieved at each split defect resonance frequency. Figure 2 presents enlarged top- and front-views of the ten defect mode shapes (*i.e.*, out-of-plane displacement fields), each demonstrating the wave behaviors at ten distinctive defect band frequencies: {56.80, 57.06 kHz}, {56.71, 57.12 kHz}, {56.86, 57.16 kHz}, {59.51, 59.85 kHz}, {60.75, and 61.45 kHz}. It is interestingly observed that the ten defect band frequencies yield ten different defect mode shapes. Moreover, the two defect mode shapes in each pair of the split bands are similar to each other except that they are in opposite phases. For example, each defect in Fig. 2a, b exhibits the same defect mode shape in Additional file 1: Fig. S4a while the defects behave in in-phase mode at the lower frequency of 56.80 kHz (Fig. 2a) and in out-of-phase mode at the higher frequency of 57.06 kHz (Fig. 2b), respectively. Likewise, in-phase behaviors are observed at the lower split defect band frequency as shown in Fig. 2c, e, g and i, whereas out-of-phase behaviors appear at the upper split frequencies as presented in Fig. 2d, f, h and j. Here, the meanings of the in-phase and out-of-phase behaviors are that the defect mode shape of each defect in Fig. 2 is polarized in the same opposite directions, respectively. It is also noteworthy that those five distinctive mode shapes are similar to the mode shapes shown in their counterpart single defect case presented in Additional file 1: Fig. S2. For instance, the monopole-like mode shape at {59.51, 59.85 kHz} in Fig. 2g, h for the double defect (4, 7) case is associated with the monopole-like mode shape at 59.65 kHz for single defect case in Additional file 1: Fig. S4d. The analysis results above suggest that the defect mode shapes are mostly maintained after the defect band splitting takes place due to the interaction between the double defects.

**Fig. 3:**
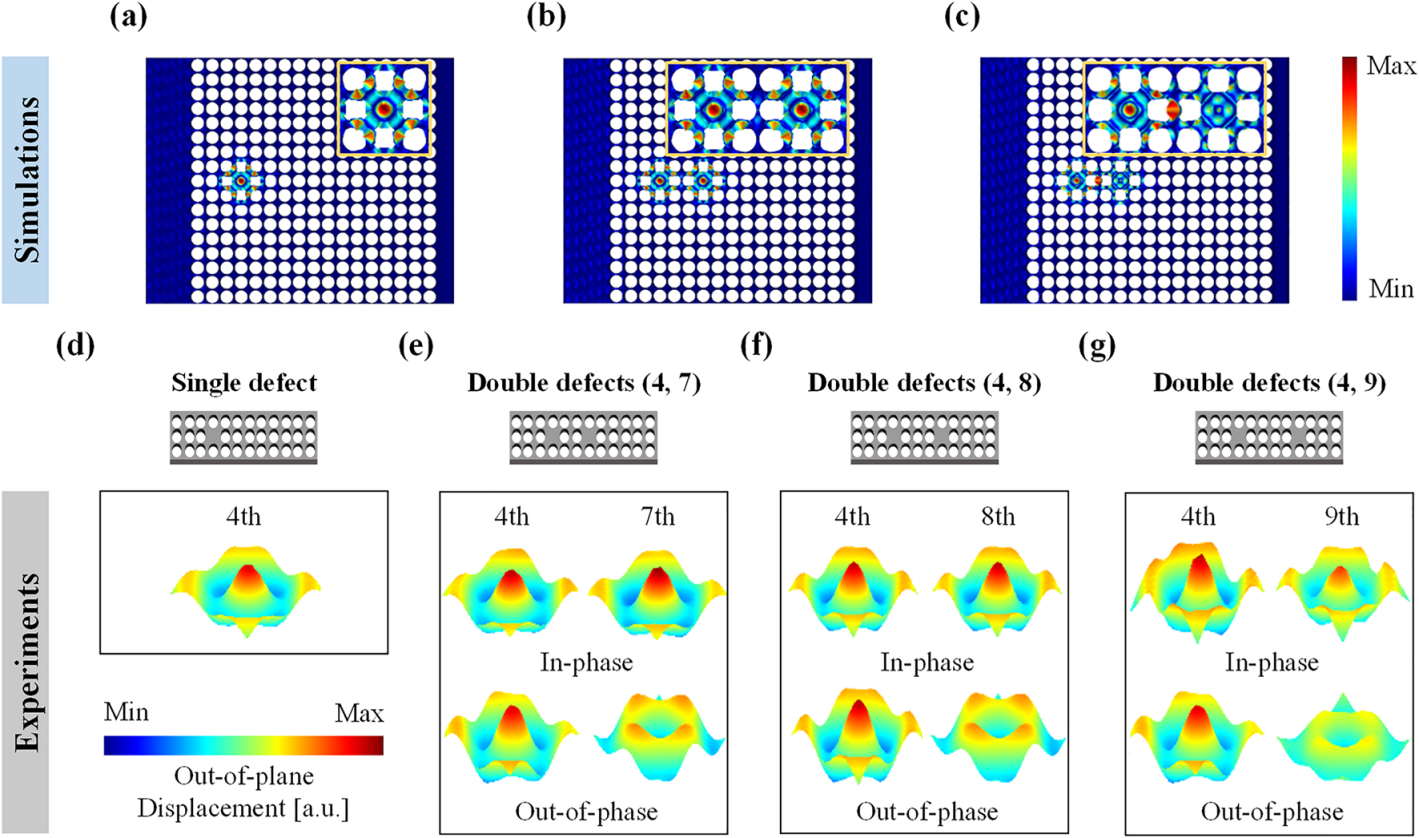
Visualization results of the in-phase and out-of-phase monopole-like defect mode shapes inside the double defects of Phononic crystals: **a**–**c** time-harmonic wave propagation behaviors in numerical simulations for the single defect case at 59.65 kHz and the double defect case at 59.51 and 59.85 kHz, respectively. **d**–**g** Experimentally scanned out-of-plane operating deflection shapes for the single defect case and the double defect case (4, 7), (4, 8), and (4, 9), respectively

Figure 3 compares the simulated and monopole-like defect mode shapes with the experimentally measured monopole-like defect mode shapes for both single- and double-defected phononic crystals. Figure 3a shows the time-harmonic analysis results of the *x*_3_-directional operating deflection shapes for the single defect case at 59.65 kHz, upon the incident plane waves from the left side (Additional file 1: Fig. S2); evanescent waves in the single defect are amplified with the monopole-like defect mode shape. Figure 3b, c present the in-phase and out-of-phase monopole-like defect mode shapes for the double defects (4, 7) at two split defect band frequencies of 59.51 kHz and 59.85 kHz, respectively. Figure 3d, e are the tilted-views of the experimentally visualized monopole-like defect mode shapes for the single and the double defects (4, 7) cases, respectively, which successfully validate the simulated results in Fig. 3a–c. In Fig. 3(e), the laser-scanned out-of-plane displacement fields in the two defected area of the double defect (4, 7) case clearly show the in-phase (top) and out-of-phase (bottom) behavior of the defects each at the lower and higher split defect band frequencies. Note that incident waves are generated at the experimentally determined defect band frequencies for each case. Detailed dynamic behaviors for the single, the double defects (4, 7) in-phase, and the double defects (4, 7) out-of-phase cases are available in Additional file 2: Video S1. Additional file 3: Video S2, Additional file 4: Video S3. We also performed the experimental visualization of the defected areas for the double defect cases (4, 8) and (4, 9) at split defect band frequencies of each case. Again, the in-phase and out-of-phase defect mode shapes are successfully observed in experimental results in Fig. 3f, g. For all three double-defected cases, the 4th defect always acts as a mechanical resonator similar to the single defect, while the defects located farther from the incident waves (*i.e.,* 7th, 8th, and 9th defects) resonate in opposite directions and result in the out-of-phase mode shapes at the higher split frequencies.

**Fig. 4:**
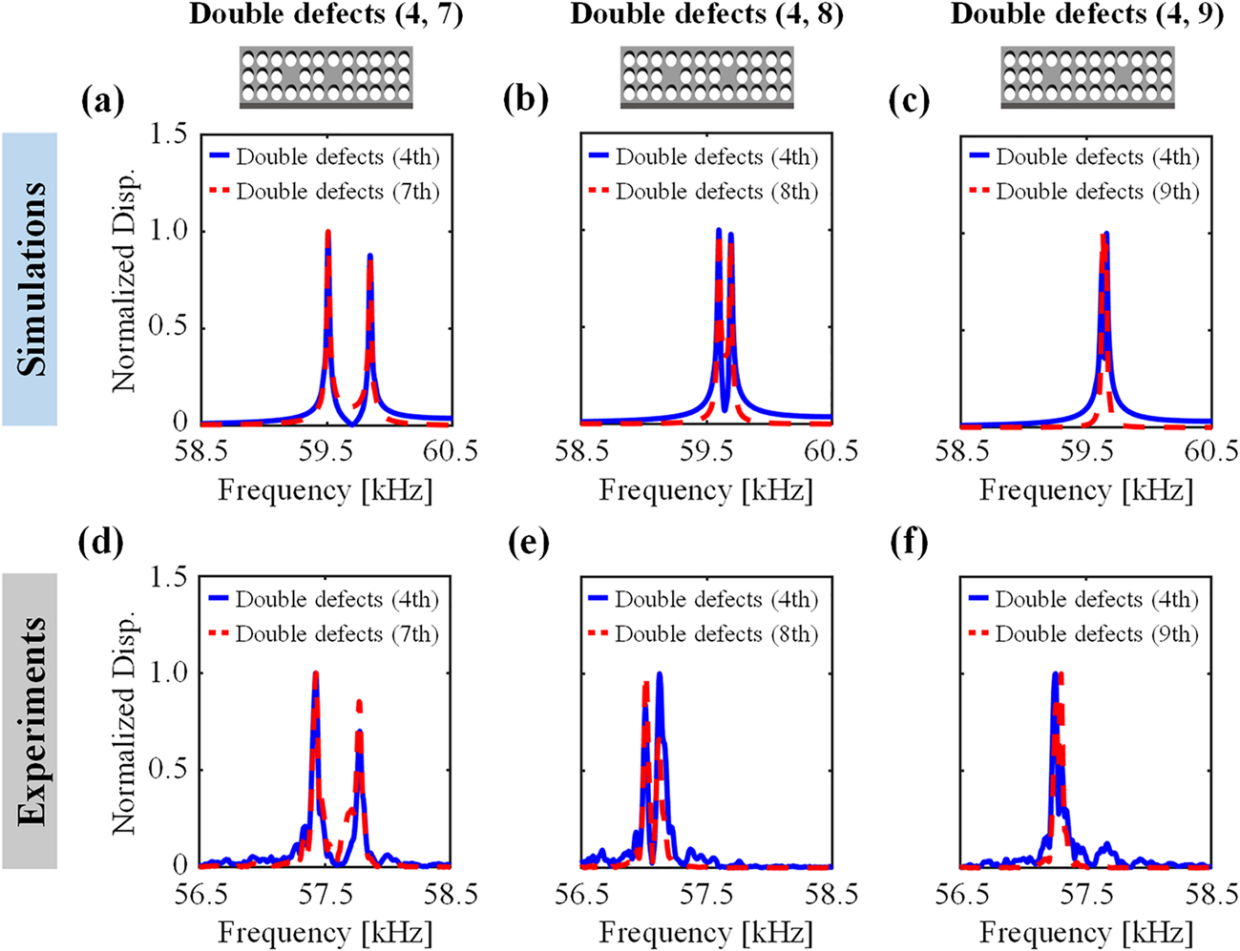
The degree of the defect band splitting is observed to decrease with increasing distance between the double defects in both simulation and experiment. **a**–**c** Numerical simulation results of the normalized out-of-plane displacement frequency response functions for three cases of double defects (4, 7), (4, 8), and (4, 9), respectively. **d**–**f** Experimental results of the normalized out-of-plane displacement frequency response functions for three cases of double defects (4, 7), (4, 8), and (4, 9), respectively

We further investigate the effects of the distance between the double defects on the degree of the splitting for the three double defect cases: (4, 7), (4, 8), and (4, 9). Figure 4 includes both the simulated and experimental results of the frequency response functions for out-of-plane displacements at the center of each defect, where the distance between the double defects increases from the left to right: Fig. 4a–c in simulation, Fig. 4d–f in experiment. In both simulation and experiment, the closer the spacing between the defects, the larger the difference between the two split frequencies because of the stronger the interaction between the localized defect modes. Note that the degree of the splitting becomes larger with decreasing spacing between the defects but that they are nonlinearly related. For quantitative comparison, Table 1 summarizes the defect band frequencies that correspond to the monopole-like defect mode shapes for cases with a single defect and three double defect combinations. The degree of the splitting can be determined by the difference between the lower and higher defect band frequencies, which are obtained as 340, 100, and 30 Hz for (4, 7), (4, 8) and (4, 9), respectively. Importantly, the decreasing gap between the peak defect band frequencies with increasing defect interspacing from (4, 7) to (4, 9) in Fig. 4a–c are in excellent qualitative and quantitative agreement with their experimental counterparts in Fig. 4d–f.

**Table 1:** Effects of the distance between the double defects on the defect band splitting along with its comparison with the single defect case

Number of defects	Defect location	Numerical simulations			Experiments		
		Defect band frequency [kHz]		Differences [Hz]	Defect band frequency [kHz]		Differences [Hz]
Single	4th	59.65		–	58.11		–
Double	(4, 7)	59.51	59.85	340	57.43	57.78	350
	(4, 8)	59.60	59.70	100	57.02	57.12	100
	(4, 9)	59.63	59.66	30	57.26	57.30	40

**Fig. 5:**
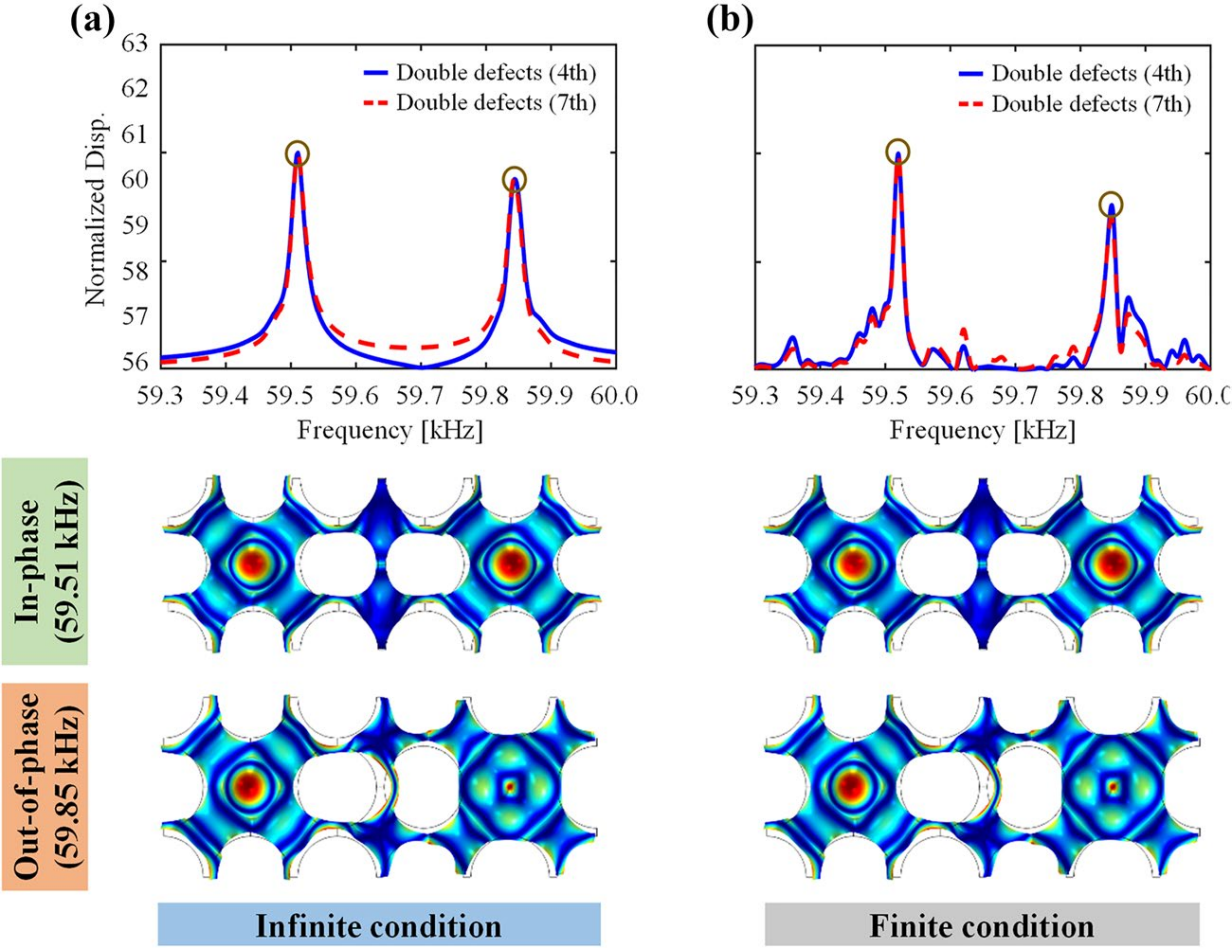
Comparison of numerical simulation results under the **a** infinite and **b** finite domain conditions: normalized out-of-plane displacement frequency response functions (top) and the *x*_3_-directional operating deflection shapes (bottom) inside the double defects (4, 7) in the phononic crystal at the peak frequencies of 59.51 and 59.85 kHz

One of the significant issues in the comparison of the simulated and experimental results is that the subject and surrounding environment in simulation should be reproduced the same as the experimental counterpart as much as possible, or vice versa. Here, we probe the boundary condition settings in numerical simulations. Applying periodic boundary condition and perfectly matched layers as illustrated in Additional file 1: Fig. S2 is quite conventional in numerical simulations and used in this work, in which “infinite domain” is assumed. However, realizing the infinite plate condition is not quite realistic in experiments. Although we fabricated the phononic crystals using the largest aluminum plate commercially available (2000 × 1000 × 2 mm^3^) to minimize the influence of the reflected waves from the plate boundaries on the results, finite condition issues still remain. Therefore, we perform the same numerical simulation under the finite domain condition (Additional file 1: Fig. S3) to examine whether the defect band frequencies can be observed in the same manner as under the infinite conditions. Figure 5a, b show the calculated frequency response functions for the normalized out-of-plane displacement under the infinite and finite domain conditions, respectively, along with their time-harmonic analysis results of the defect mode shapes. The overall trend of the displacement frequency response functions and the defect mode shapes seems similar, regardless of the boundary conditions. Indeed, the main two distinctive peaks of the split defect band frequencies appear in the same position at 59.51 and 59.85 kHz under both conditions. The minor peaks that appear around the main peaks in the numerically calculated frequency response function plots in Fig. 5b indicate that there still exist the effects of the wave reflection from the edges of the plate, as expected. These minor peaks are also observed in the experiments as shown in Fig. 4d–f. These minor peaks, however, do not affect the overall trend in the defect band splitting, as well as defect mode shapes at all. It can thus be concluded that not only the defect band splitting but also the energy localization phenomenon is reasonably simulated and predicted regardless of whether the structure is under the infinite or finite domain conditions.

**Fig. 6:**
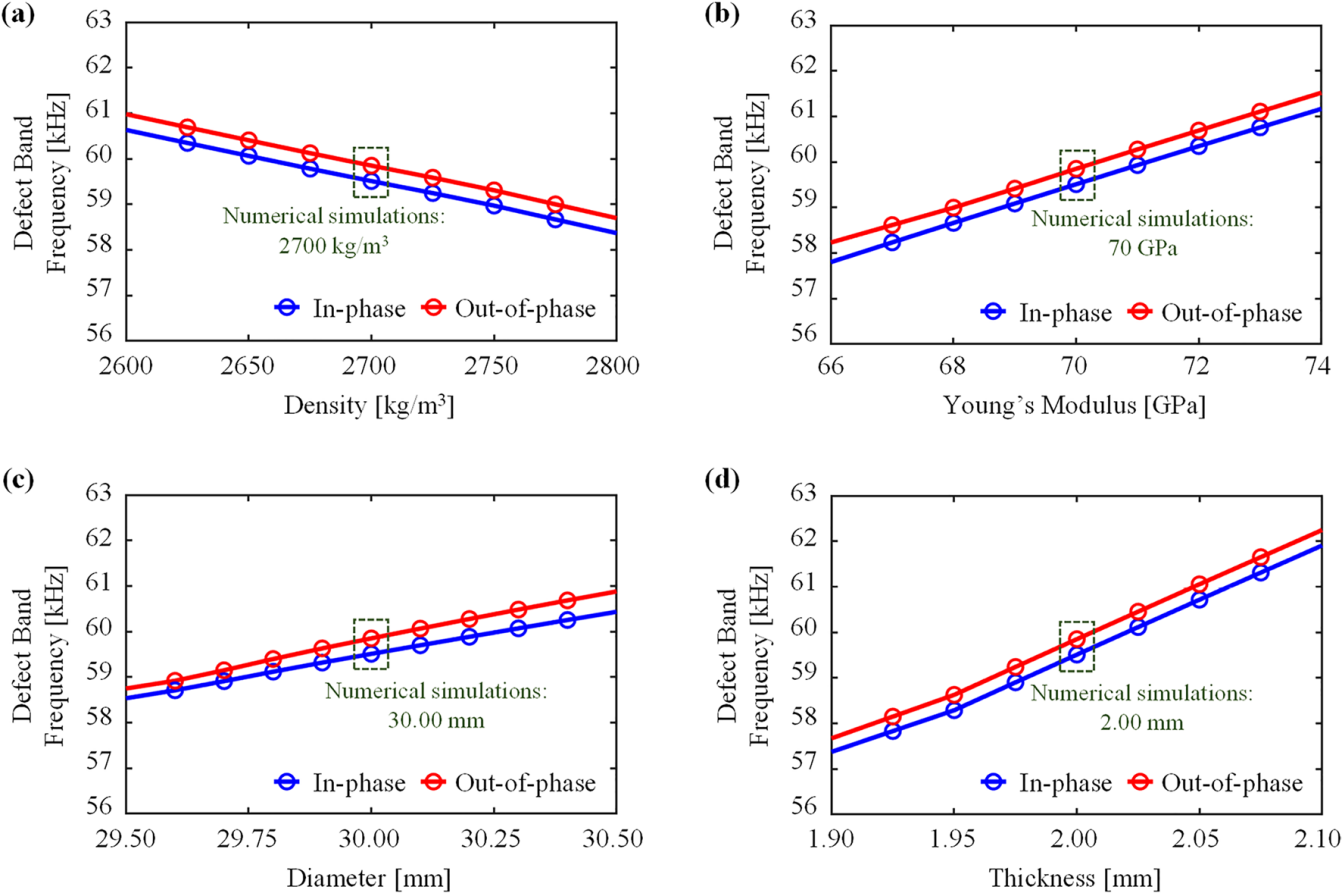
Sensitivity analysis results of the defect band frequencies that correspond to the in-phase and out-of-phase monopole-like defect modes in terms of material properties and geometric variation. **a** Effects of the mass density of the aluminum plate, ranging from 2600 to 2800 kg/m^3^. **b** Effects of Young’s modulus of the aluminum plate, ranging from 66 to 74 GPa. **c** Effects of the diameter of the circular hole, ranging from 29.50 to 30.50 mm. **d** Effects of the thickness of the unit cell, ranging from 1.90 to 2.10 mm

Another possible source of discrepancy between simulation and experiment is various physical uncertainties arising from variability in the material properties and geometric dimensions in practice (*e.g.,* fabrication). Statistically, these kinds of uncertainties are called an aleatory uncertainty, which refers to the objective and irreducible uncertainty that arises from inherent randomness in engineering products [62]. It is observed in Fig. 4d–f that the experimentally obtained main peak frequencies shifted by 1.5–2 kHz from the simulated peaks. To identify the origin of this shift in the absolute value in the peak frequencies from simulation and experiment, we investigate the sensitivity of defect band frequencies to the physical uncertainties. Figure 6a–d present the defect band results for a phononic crystal with double defects (4, 7) while varying the mass density and Young’s modulus of the aluminum plate, the diameter of the circular hole, and thickness of the unit cell, respectively. When one parameter is varied, the other parameters are all fixed. For instance, Fig. 6a is the result of the mass density variation, ranging from 2600 to 2800 kg/m^3^, while the other quantities are fixed as 70 GPa, 30, and 2 mm. The defect resonant frequencies decrease with increasing mass density whereas they increase with increasing Young’s modulus, as demonstrated in Fig. 6a, b. These trends are consistent with the physical observations in vibrations of a structure’s mass and stiffness effects on a natural frequency. As shown in Fig. 6c, d, the defect band frequencies increase as either diameter of the circular hole or thickness increases. Note that the defect band frequencies are relatively more sensitive to the thickness of the plate with sharper slope in Fig. 6d than the diameter (Fig. 6c). When diameter increases, the degree of the defect band splitting also increases in accordance with increasing diameter. Indeed, the uncertainties of the material properties and geometric dimensions can lead to significant variations of defect band frequencies. Considering that the resolution of the laser cutting used for specimen fabrication is around 0.1 mm in this work and that there is inevitable material property variation at the production level, it can give rise to variation in both material and geometric uncertainties. Therefore, the sensitivity analysis results unambiguously explain the shift in the peak frequency position in the experiment in Fig. 4d–f from the simulation within quite a reasonable range.

## Conclusion

In summary, we demonstrated both numerical simulations and experimental validation of defect band splitting for a phononic crystal under A_0_ Lamb waves. We explored the effects of distance between double defects on the degree of the splitting. The designed phononic crystal used in this study was a 17 × 17 repetitive array of typical unit cells, which consisted of an aluminum square lattice with a circular hole, while single or double defects (4, 7), (4, 8), (4, 9) were introduced by not puncturing some holes in unit cells. Even though the physical uncertainties of material properties and geometric dimensions inherently existed in the fabricated aluminum plates, the degrees of defect band splitting for the three cases of the double defects in experiments were in excellent agreement with those in numerical simulations. It was confirmed that the degree of defect band splitting became increasing as the distance between the double defects was farther. Furthermore, for all cases, the experimental scanning successfully visualized in-phase and out-of-phase monopole-like defect mode shape behaviors at each of the split defect bands, respectively. From the perspective of boundary conditions and physical variability of material properties and geometry, the slight discrepancy between the results obtained by the numerical simulations and experiments was thoroughly analyzed. As one of the engineering applications of defect band splitting, further numerical and experimental investigations on widening bandwidths of piezoelectric energy harvesting via combining phononic crystals with single and double defects will be presented in the near future.

## Supplementary Information

**Additional file 1. Fig. S1.** A schematic illustration of a phononic crystal having **a** single defect or **b** double defects with a borderline of the 1st irreducible Brillouin zone. **Fig. S2.** Boundary and loading conditions for time-harmonic analysis in numerical simulations. Perfectly matched layers and periodic boundary conditions are set as “infinite” boundary conditions, where transversely excitation of the thin plate for generating A_0_ Lamb waves is applied as loading conditions. **Fig. S3.** A schematic diagram of the “finite” boundary condition for numerical analysis that matches with the experimental environment: a large aluminum plate having geometric dimension of 2000 × 1000 × 2 mm^3^ with the 17 × 17 phononic crystal supercell introduced from a distance of 1000 mm from the left end. **Fig. S4.** Defect mode shapes (out-of-plane displacement fields) for the phononic crystal with the single defect at the defect band frequencies that correspond to the five defect bands presented in Fig. 1b: **a** 56.83 kHz, **b** 56.87 kHz, **c** 56.94 kHz, **d** 59.65 kHz (monopole-like defect mode shape), and **e** 61.00 kHz. **Fig. S5.** Experimental setups for elastic wave generation (PZT transducers, function generator, and power amplifier) and elastic wave visualization (laser Doppler vibrometer, data acquisition, and controller). **a** A schematic illustration of the experimental setup with the fabricated aluminum plate. **b** A photo of the laboratory environment with the experimental testbed and the fabricated double defected phononic crystal specimen. **Fig. S6.** Time-domain data of the out-of-plane displacements measured at the center of each defect for **a** the single defect and for **b** the 4th defect and **c** the 7th of the double defects. **Fig. S7.** Snapshots of the oscillating defect mode shapes obtained using scanning laser Doppler vibrometer. **a** Visualizing the in-phase monopole-like defect mode shape in the isometric view with (1st) and without (2nd) the host plate and in the top view with (3rd) and without (4th) the host plate. **b** Visualizing the out-of-phase monopole-like defect mode shape in the isometric view with (1st) and without (2nd) the host plate and in the top view with (3rd) and without (4th) the host plate.

**Additional file 2.** Experimentally visualized energy-localized behavior (monopole-like defect mode shape) within the defect for the single defect case at the peak frequency of 58.11 kHz.

**Additional file 3. **Experimentally visualized energy-localized behaviors (in-phase monopole-like defect mode shapes) within the defects for the double defect (4, 7) case at the peak frequency of 57.43 kHz.

**Additional file 4. **Experimentally visualized energy-localized behaviors (out-of-phase monopole-like defect mode shapes) within the defects for the double defect (4, 7) case at the peak frequency of 57.78 kHz.

## Data Availability

The datasets used and/or analyzed during the current study are available from the corresponding author on reasonable request.
