# Supplementary material for: Double defects-induced elastic wave coupling and energy localization in a phononic crystal
Source: Nano Converg. 2021 Sep 16;8:27. doi: 10.1186/s40580-021-00277-4 (PMC8446117; doi:10.1186/s40580-021-00277-4)
Supplement: Supplementary file 1 — Additional file 1. Fig. S1. A schematic illustration of a phononic crystal having a single defect or b double defects with a borderline of the 1st irreducible Brillouin zone. Fig. S2. Boundary and loading conditions for time-harmonic analysis in numerical simulations. Perfectly matched layers and periodic boundary conditions are set as “infinite” boundary conditions, where transversely excitation of the thin plate for generating A0 Lamb waves is applied as loading conditions. Fig. S3. A schematic diagram of the “finite” boundary condition for numerical analysis that matches with the experimental environment: a large aluminum plate having geometric dimension of 2000 × 1000 × 2 mm3 with the 17 × 17 phononic crystal supercell introduced from a distance of 1000 mm from the left end. Fig. S4. Defect mode shapes (out-of-plane displacement fields) for the phononic crystal with the single defect at the defect band frequencies that correspond to the five defect bands presented in Fig. 1b: a 56.83 kHz, b 56.87 kHz, c 56.94 kHz, d 59.65 kHz (monopole-like defect mode shape), and e 61.00 kHz. Fig. S5. Experimental setups for elastic wave generation (PZT transducers, function generator, and power amplifier) and elastic wave visualization (laser Doppler vibrometer, data acquisition, and controller). a A schematic illustration of the experimental setup with the fabricated aluminum plate. b A photo of the laboratory environment with the experimental testbed and the fabricated double defected phononic crystal specimen. Fig. S6. Time-domain data of the out-of-plane displacements measured at the center of each defect for a the single defect and for b the 4th defect and c the 7th of the double defects. Fig. S7. Snapshots of the oscillating defect mode shapes obtained using scanning laser Doppler vibrometer. a Visualizing the in-phase monopole-like defect mode shape in the isometric view with (1st) and without (2nd) the host plate and in the top view with (3rd) and without (4th) the hos [file 40580_2021_277_MOESM1_ESM.docx]

Double defects-induced elastic wave coupling and energy localization in a phononic crystal

Soo-Ho Jo^1,2^, Yong Chang Shin^1^, Wonjae Choi^3^, Heonjun Yoon^4^, Byeng D. Youn^1,2,5,*^, and Miso Kim^6,*^

^1^ Department of Mechanical Engineering, Seoul National University, Seoul 08826, Republic of Korea

^2^ Institute of Advanced Machines and Design, Seoul National University, Seoul 08826, Republic of Korea

^3^ AI Metamaterial Research Team, Korea Research Institute of Standards and Science, Daejeon 34113, Republic of Korea

^4^ School of Mechanical Engineering, Soongsil University, Seoul 06978, Republic of Korea

^5^ OnePredict Inc., Seoul 06160, Republic of Korea

^6^ School of Advanced Materials Science & Engineering, Sungkyunkwan University, Suwon 16419, Republic of Korea

^*^ Correspondence to: Byeng D. Youn (Tel: +82-2-880-1919, E-mail: bdyoun@snu.ac.kr) and Miso Kim (Tel: +82-31-290-7415, E-mail: smilekim@skku.edu)

**Fig. S1.** A schematic illustration of a phononic crystal having (a) single defect or (b) double defects with a borderline of the 1^st^ irreducible Brillouin zone.

**Fig. S2**. Boundary and loading conditions for time-harmonic analysis in numerical simulations. Perfectly matched layers and periodic boundary conditions are set as “infinite” boundary conditions, where transversely excitation of the thin plate for generating A_0_ Lamb waves is applied as loading conditions.

**Fig. S3**. A schematic diagram of the “finite” boundary condition for numerical analysis that matches with the experimental environment: a large aluminum plate having geometric dimension of 2000×1000×2 mm^3^ with the 17×17 phononic crystal supercell introduced from a distance of 1000 mm from the left end.

**Fig. S4.** Defect mode shapes (out-of-plane displacement fields) for the phononic crystal with the single defect at the defect band frequencies that correspond to the five defect bands presented in Fig. 1(b): (a) 56.83 kHz, (b) 56.87 kHz, (c) 56.94 kHz, (d) 59.65 kHz (monopole-like defect mode shape), and (e) 61.00 kHz.

**Fig. S5.** Experimental setups for elastic wave generation (PZT transducers, function generator, and power amplifier) and elastic wave visualization (laser Doppler vibrometer, data acquisition, and controller). (a) A schematic illustration of the experimental setup with the fabricated aluminum plate. (b) A photo of the laboratory environment with the experimental testbed and the fabricated double defected phononic crystal specimen.

**Fig. S6.** Time-domain data of the out-of-plane displacements measured at the center of each defect for (a) the single defect and for 9b) the 4^th^ defect and (c) the 7^th^ of the double defects.

**
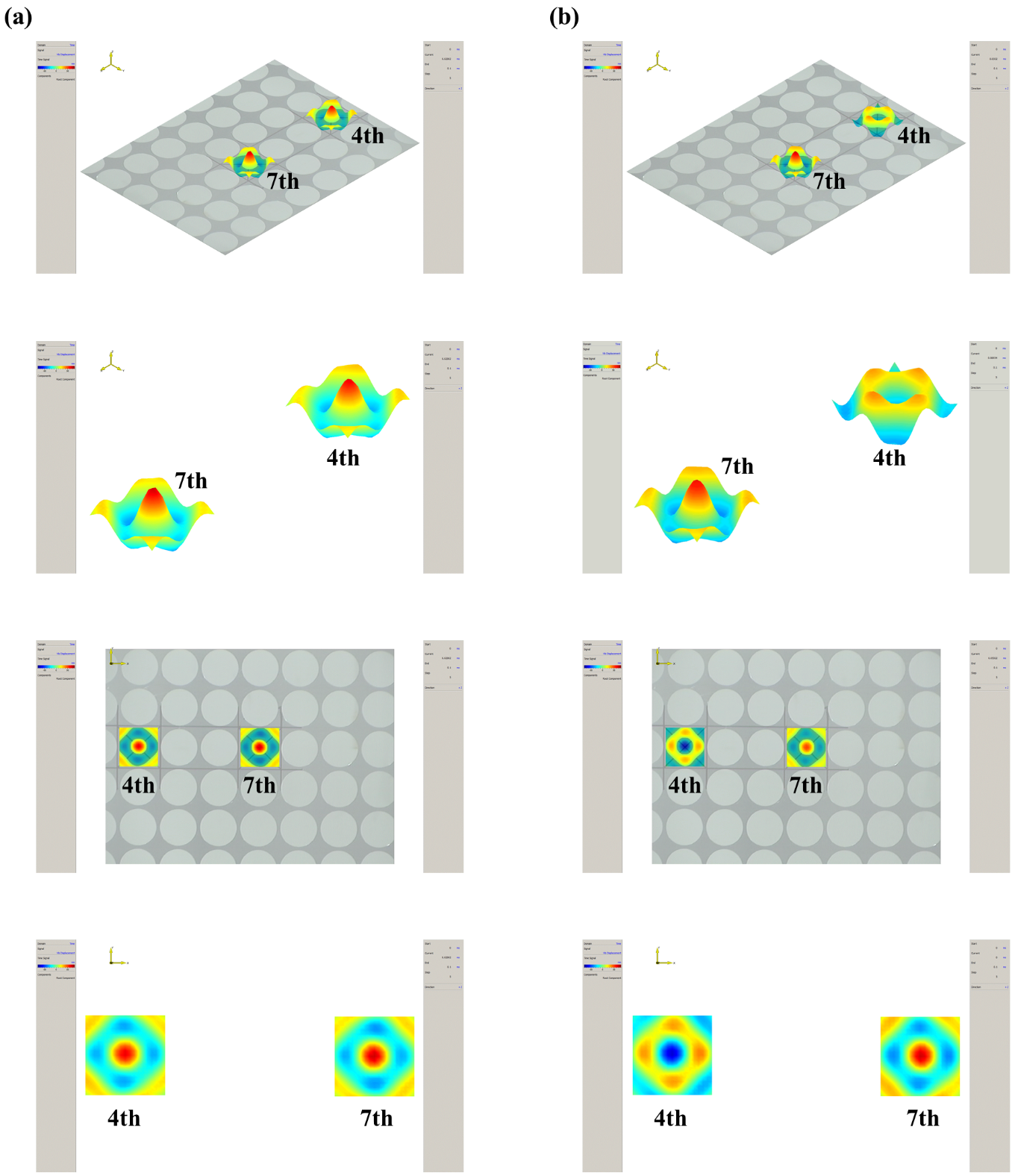
**

**Fig. S7**. Snapshots of the oscillating defect mode shapes obtained using scanning laser Doppler vibrometer. (a) Visualizing the in-phase monopole-like defect mode shape in the isometric view with (1^st^) and without (2^nd^) the host plate and in the top view with (3^rd^) and without (4^th^) the host plate. (b) Visualizing the out-of-phase monopole-like defect mode shape in the isometric view with (1^st^) and without (2^nd^) the host plate and in the top view with (3^rd^) and without (4^th^) the host plate.
